# Vitamin B12 Deficiency Manifesting As Pancytopenia, Lymphadenopathy, and Fever: A Clinical Mimic of Hematologic Malignancies

**DOI:** 10.7759/cureus.64676

**Published:** 2024-07-16

**Authors:** Eunhee Choi, Gabriela Galicia Garcia, Krishna Kishore Anna, Maria F Albuja Altamirano, Laverne Yip, Jaha Oh, Jung-Hyun Lee

**Affiliations:** 1 Internal Medicine, NYC Health and Hospital/Lincoln, New York, USA; 2 Nephrology, New York Presbyterian/Weill Cornell Medical Center, New York, USA; 3 Neurology, Maimonides Medical Center, New York, USA; 4 Neurology, State University of New York Downstate Health Sciences University, New York, USA; 5 Neurology, Kings County Hospital Center, New York, USA

**Keywords:** vitamin b12 deficiency, pancytopenia, mimic hematologic malignancy, cobalamin, pernicious anemia, cobalamin deficiency

## Abstract

Pancytopenia is a complex medical condition characterized by decreased levels of red blood cells (RBCs), white blood cells (WBCs), and platelets (PLTs). It can arise from impaired production, peripheral destruction, or a combination of both. The causes of pancytopenia range from reversible factors like infections and medication reactions to irreversible conditions. Vitamin B12 deficiency is a notable reversible cause that can take years to manifest in adults due to stored reserves. However, deficiencies caused by impaired absorption, especially due to the lack of intrinsic factors (IFs), can lead to rapid deterioration within two to five years.

A healthy 39-year-old male with an athletic lifestyle presented with symptoms such as dizziness, nausea, vomiting, palpitations, and fainting over a few days. These symptoms were preceded by weeks of persistent body aches, headaches, weakness, daily fevers, chills, and night sweats. Vital signs were stable. The physical examination revealed conjunctival pallor and lymphadenopathy in the submandibular and superficial cervical regions. Initial blood tests showed normocytic anemia (Hgb 4.9, MCV 80), leukopenia (2.99), thrombocytopenia (142), and elevated liver enzymes (AST 199, ALT 96, and total bilirubin of 2.04). The peripheral smear showed tear-drop cells and hypochromic cells.

The initial impression was hematologic malignancy, including but not limited to leukemia, lymphoma, or myelofibrosis given clinical findings such as B-symptoms like night sweats, neck lymphadenopathy, and subjective daily fever, along with pancytopenia.

The patient received a bolus of normal saline and a transfusion of two units of packed RBCs. CT scans of the chest, abdomen, and pelvis showed no adenopathy or splenomegaly. Although initial clinical assessment pointed toward a potential hematologic malignancy, comprehensive testing, including SPEP, reticulocyte count/fraction, serum folate, and serum vitamin B12, revealed only severe vitamin B12 deficiency, with a level of less than 150, with the presence of IF antibodies.

Treatment involved intensive in-patient vitamin B12 injections followed by a detailed outpatient regimen. The patient completed a daily dose of vitamin B12 injections for seven consecutive days, followed by weekly injections for the next four weeks. Subsequent laboratory results demonstrated an increase in WBC count to 8.39, Hgb level to 13.2, and PLT count of 249, indicating a continued positive response to the vitamin B12 replacement therapy.

In summary, pancytopenia poses a diagnostic challenge that demands careful evaluation of patient data and comprehensive testing. Vitamin B12 deficiency, which encompasses pernicious anemia (PA), is among the reversible factors to consider. This aspect holds significance before opting for more invasive measures like a bone marrow biopsy. Nutritional deficiencies need to be considered first as differentials in pancytopenia, even in the absence of typical signs of vitamin B12 deficiency (like macrocytosis and hypersegmented neutrophils) and in the presence of compelling clinical indications pointing to a hematologic malignancy.

## Introduction

Pancytopenia is a medical condition characterized by decreased levels of all three primary blood cell types: red blood cells (RBCs), white blood cells (WBCs), and platelets (PLTs). The etiological factors contributing to pancytopenia can be broadly classified into three categories: impaired production, which includes bone marrow failure disorders and marrow infiltration disorders; peripheral destruction; and a mixed type [1]. These causes can also be categorized into reversible and irreversible, indicating the potential for recovery or the permanence of the condition, respectively.

Reversible causes of pancytopenia include viral infections such as Epstein-Barr virus or Parvovirus B19, certain medications, chronic alcohol use disorder, and deficiencies in vitamins or minerals such as vitamin B12, folate, or iron. This report focuses specifically on vitamin B12 deficiency.

Vitamin B12, or cobalamin, is a water-soluble vitamin derived from animal-based sources like red meat, dairy, and eggs. Its absorption in the terminal ileum relies on the intrinsic factor (IF), a glycoprotein produced by the stomach's parietal cells. Once absorbed, vitamin B12 is a crucial cofactor for key enzymes involved in the synthesis of DNA, fatty acids, and myelin [2].

As the body requires relatively low daily supplements of vitamin B12 compared to its stores, a deficiency often takes years to manifest in adults. However, the absence of IF disrupts the absorption of both free and food-bound cobalamin, as well as the daily reabsorption of approximately half of the 1.4 μg of biliary cobalamin. Persistent malabsorption accelerates the progression of the deficiency, with clinical repercussions potentially appearing within two to five years [3].

Vitamin B12 deficiency can cause a range of hematologic and neurological symptoms. Macrocytic anemia is a common manifestation, with initial signs often including fatigue and pallor. Increased hemolysis due to defective erythropoiesis can lead to jaundice, serving as a potential indicator of the condition. Other early symptoms may include peripheral neuropathy, glossitis, diarrhea, headaches, and neuropsychiatric disturbances [2].

This case report discusses a young male who presented with symptoms including pancytopenia, lymphadenopathy, and fever-clinical signs often associated with hematologic malignancy. However, further evaluation revealed his condition was due to vitamin B12 deficiency.

## Case presentation

A 39-year-old male, an ex-smoker with no significant past medical history aside from a stab wound in 2014 treated with exploratory laparoscopy and repair of gastric wall lacerations, presented with dizziness, nausea, vomiting, diarrhea, palpitations, and syncope over the past few days. Over the past month, he experienced persistent body aches, headaches, weakness, daily fevers, chills, and night sweats. He also reported a previous episode of right neck swelling with lymphadenopathy and tongue swelling, which were treated as cellulitis and presumed to be an allergic reaction at another hospital. He denied any bleeding, recent exposure to sick individuals, travel, housing changes, incarceration, alcohol consumption, or illicit drug use. Despite using over-the-counter Naproxen, his symptoms persisted.

The patient's vital signs were stable: blood pressure was 142/82 mm Hg, heart rate was 80 bpm, respiratory rate was 16 breaths/min, and oxygen saturation was 100% on room air. Orthostatic hypotension was not present. During the physical examination, the patient was awake, alert, and oriented, but appeared anxious and distressed. Non-tender, 1-2 cm lymphadenopathy was noted in the bilateral submandibular and superficial cervical regions. There were no oral lesions or jaundice. Heart sounds were normal, and lung auscultation was clear. The abdomen was soft, without organomegaly.

**Figure 1:**
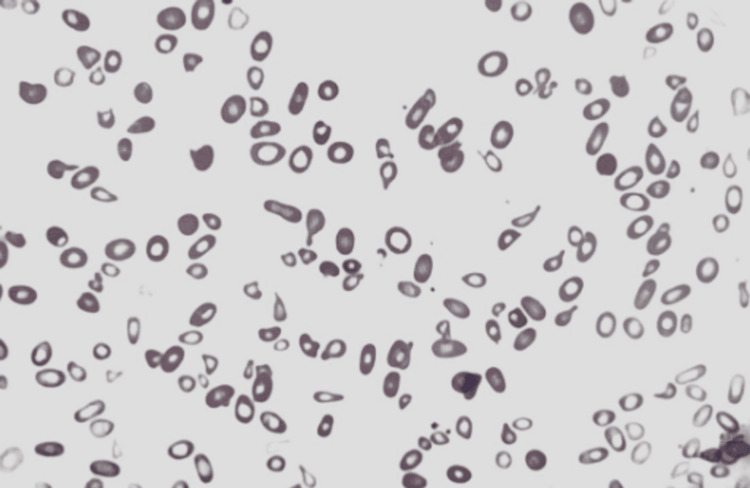
PBS: teardrop cells and hypochromic cells PBS, peripheral blood smear

Initial blood work revealed pancytopenia with a hemoglobin (Hgb) level of 4.9 g/dL and a mean corpuscular volume (MCV) of 80 fL, a WBC count of 2.99x10^3^/µL, RBC count of 1.85x10^6^/µL and thrombocytopenia with a PLT count of 142x10^3^/µL. Lactate dehydrogenase (LDH) was significantly elevated at 10,953 U/L. Liver function tests indicated an aspartate aminotransferase (AST) level of 199 U/L, an alanine aminotransferase (ALT) level of 96 U/L, and a total bilirubin level of 2.04 mg/dL with direct bilirubin at 0.3 mg/dL. Iron was 121 µg/dL, total iron-binding capacity (TIBC) was 304 µg/dL, iron saturation was 40%, ferritin was 983 ng/mL, and transferrin was 231 mg/dL. The peripheral blood smear (PBS) showed teardrop cells and hypochromic cells (Figure 1).

Electrocardiography showed normal sinus rhythm, and a chest X-ray was unremarkable. Given the laboratory and clinical findings, the initial differential diagnosis focused on hematologic malignancies, including leukemia, lymphoma, and myelofibrosis. While flow cytometry and bone marrow biopsy were considered necessary to definitively rule out these conditions, further non-invasive workups were conducted first to narrow down the diagnosis and confirm the underlying etiology while patients received Pantoprazole, fluid, and two units of packed RBCs. 

The results included a reticulocyte count of 0.32%, an absolute reticulocyte count of 0.0069x10⁶/µL, an immature reticulocyte fraction (IRF) of 10.4%, and haptoglobin was less than 20 mg/dL. The serum folate level was 16.5 ng/mL, while the vitamin B12 level was critically low at less than 150 pg/mL, with elevated methylmalonic acid (MMA) (21,600 nmol/L) and homocysteine (HC) levels (276.3 µmol/L), further supporting a diagnosis of vitamin B12 deficiency. Other tests, including serum protein electrophoresis (SPEP), hepatitis serologies, and HIV tests, were negative. CT scans of the chest, abdomen, and pelvis showed no significant findings, with no adenopathy or splenomegaly.

Considering the likely vitamin B12 deficiency causing pancytopenia, treatment with intramuscular injections of cyanocobalamin at 1,000 mcg per dose was initiated. However, a bone marrow biopsy with cytogenetic analysis was planned in case pancytopenia did not resolve with vitamin supplementation. After administering two doses, the patient was discharged and followed by an outpatient hematology clinic. The presence of IF antibodies (233.2 AU/mL) was noted. Lymphadenopathy also resolved, likely due to a transient upper respiratory infection in the setting of leukopenia. The further treatment regimen included a daily dose of vitamin B12 injection for seven consecutive days, followed by weekly injections for the next four weeks, and then monthly injections.

Following the seventh dose of cyanocobalamin injection, the patient reported symptomatic improvement. Laboratory results showed a WBC of 3.85x10^3^/µL, RBC of 3.23x10^6^/µL, Hgb level of 8.5 g/dL, MCV of 87.9 fL, hematocrit of 28.4%, PLT count of 383x10^3^/µL, and LDH of 2458 U/L. The vitamin B12 level was 1323 pg/mL.

Upon completing the four weekly doses, laboratory results demonstrated further improvement. The WBC increased to 8.39x10³/µL, Hgb level rose to 13.2 g/dL, MCV adjusted to 79.3 fL, and PLT count was 249x10³/µL, indicating a continued positive response to the vitamin B12 replacement therapy (Table 1).

**Table 1:** Hematologic response with cyanocobalamin injection WBC, white blood cells; PLT, platelet; Hgb, hemoglobin; MCV, mean corpuscular volume; LDH, lactate dehydrogenase

Lab	Units	Day 1	Day 2	Day 3	Day 11	Day 42
WBC	x10^3^/mcL	2.99	2.13	2.3	3.85	8.39
HGB	g/dL	4.9	7.4	7.8	8.5	13.2
MCV	fL	80.0	80.4	82.7	87.9	79.3
PLT COUNT	x10^3^/mcL	142	88	74	383	249
LDH	U/L	10953	-	-	2458	-

## Discussion

This discussion will outline a systematic approach to diagnosing pancytopenia, providing clinicians with a structured diagnostic algorithm. It will then explore identifying vitamin B12 deficiency, addressing challenges in interpreting serum B12 levels, and emphasizing the importance of clinical context in diagnosis. Various hematological and clinical manifestations of vitamin B12 deficiency will be presented, offering insights to aid in early recognition. Finally, the discussion will focus on pernicious anemia (PA), detailing its diagnostic criteria and current management strategies.

Pancytopenia workup algorithm

**Figure 2:**
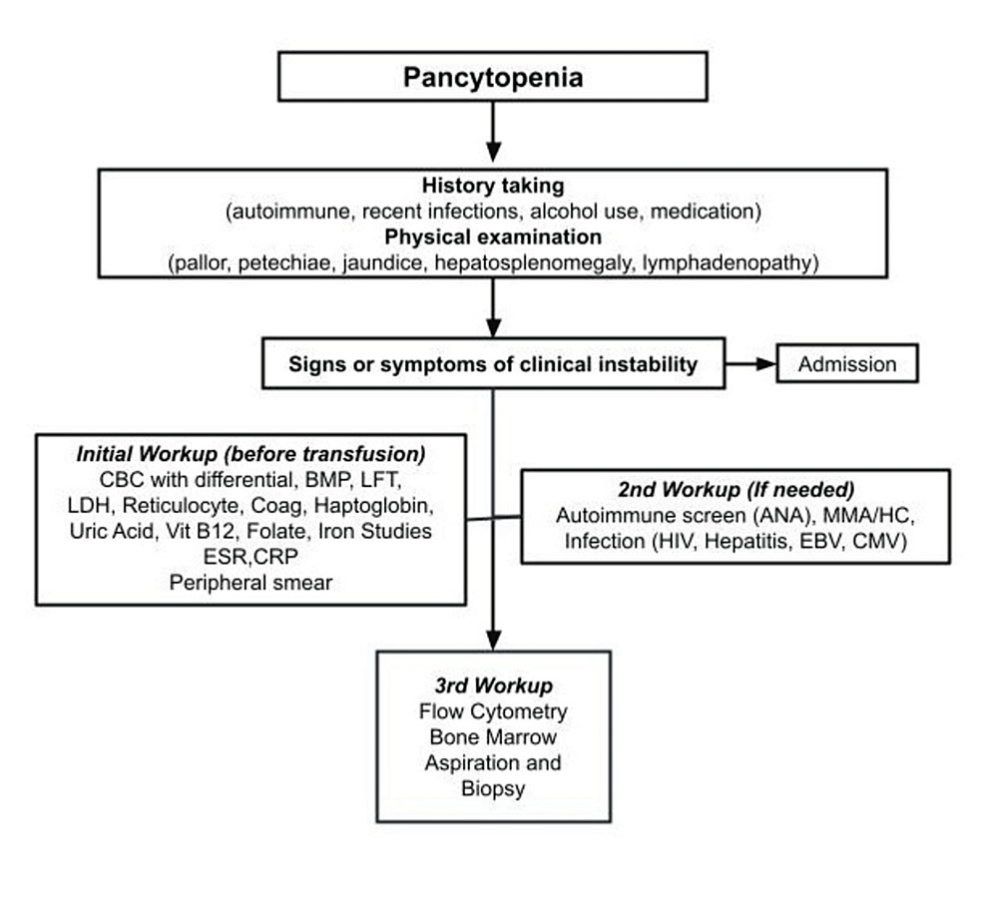
Pancytopenia workup algorithm

Pancytopenia, a frequent cause of hematology consultations, is associated with various medical conditions. A comprehensive history and physical examination are crucial in narrowing down potential causes and guiding specific diagnostic studies. An algorithm has been developed to assist physicians in diagnosing pancytopenia (Figure 2). The key to both diagnosis and patient management lies in determining whether the pancytopenia results from a production disorder, a consumption disorder, or a combination of both [1].

Vitamin B12 deficiency: interpretation, diagnosis, and pitfalls in assessment

Accurate interpretation of vitamin B12 levels is essential for diagnosis. Serum B12 levels are categorized as follows: above 300 pg/mL is considered normal, between 200 and 300 pg/mL is borderline (potentially requiring additional enzymatic testing), and below 200 pg/mL is classified as deficient.

However, it is crucial to recognize that serum cobalamin levels below 200 ng/L are frequently observed, especially in elderly populations. Approximately 22% to 30% of these low levels may be falsely low. Consequently, a low serum cobalamin (<200 or 250 ng/L) should not be automatically interpreted as diagnostic for cobalamin deficiency, particularly in asymptomatic patients with normal hematological profiles [3]. Several factors can contribute to falsely low serum cobalamin levels, including folate deficiency, oral contraceptive use, pregnancy, and multiple myeloma [3,4]. Serum cobalamin levels can be artificially elevated in patients with alcoholism, liver disease, or cancer because of decreased hepatic clearance of transport proteins and resultant higher circulating levels of vitamin B12 [5]. Accurate diagnosis requires integrating clinical assessment with laboratory results.

The initial diagnostic approach involves documenting clinical and laboratory findings and establishing their connection to cobalamin deficiency. When the clinical picture is equivocal, serum MMA or plasma HC can provide valuable insights. Obtaining all samples before initiating cobalamin treatment is essential, as cobalamin levels rise immediately, and metabolite levels improve within several days, rendering subsequent measurements less informative [3].

Hematological manifestations of vitamin B12 deficiency

Vitamin B12 deficiency commonly causes bone marrow suppression, potentially affecting all blood cell lines due to impaired DNA synthesis and repair processes. The most frequent manifestation is megaloblastic anemia. The abnormal erythropoiesis can lead to other abnormal laboratory findings, such as decreased haptoglobin levels, high LDH levels, elevated reticulocyte count, and elevated indirect bilirubin.

In typical vitamin B12 deficiency, the MCV usually increases before a significant drop in Hgb levels occurs. However, MCV can often stay within the reference range, particularly if there is concurrent iron deficiency, thalassemia, or chronic illness present [6]. In our case, iron deficiency anemia was ruled out, but a thalassemia workup with Hgb electrophoresis was not performed.

A PBS may show macro-ovalocytes and hypersegmented neutrophils - at least one neutrophil with six lobes or more anisopoikilocytosis. Although hypersegmented neutrophils are often considered a hallmark of megaloblastic anemia, they are not specific, as seen in other types of anemia, like iron deficiency anemia. Hypersegmented neutrophils typically precede macrocytosis and anemia; however, in advanced diseases, they may be rare or absent like the current case [4,7]. Teardrop erythrocytes are particularly prominent in patients with myelofibrosis with myeloid metaplasia and occur frequently in patients with other infiltrative disorders of the bone marrow, such as leukemia and metastatic carcinoma [8]. However, teardrop cells can also be observed in the advanced macro-ovalocytes [9].

Vitamin B12 facilitates the synthesis of methionine from HC and enables the conversion of methylmalonyl coenzyme A to succinyl coenzyme A. These processes can be assessed by measuring metabolite levels, with diagnostic cutoffs established at >260 nmol/L for MMA and >12 μmol/L for HC [10]. MMA exceeding 1000 nmol/L warrants immediate and thorough clinical attention, as it strongly indicates a severe deficiency or metabolic disorder related to vitamin B12 function [3]. However, renal insufficiency can lead to elevated MMA levels and decreased HC concentrations [11].

Pernicious anemia: diagnostic approach

Vitamin B12 deficiency can be caused by medications, drugs, malabsorption, or malnutrition (Table 2) [12,13]. One of the causes is PA, an autoimmune disease characterized by chronic atrophic gastritis and defective absorption of cobalamin from the terminal ileum due to interference by the anti-intrinsic factor (anti-IF) antibodies. As PA without treatment can lead to various symptoms with irreversible neurological sequelae (Table 3), It is recommended to test for IF antibodies in a patient with suspected cobalamin deficiency [3,7,14].

**Table 2:** Causes of vitamin B12 deficiency PA, pernicious anemia; IF, intrinsic factor

Causes		Effect
Drugs	Metformin	Decreased absorption of B12
	Proton pump inhibitors	Defective release of B12 from food
	Histamine H2-receptor antagonist	Defective release of B12 from food
	Nitrous oxide	Inactivation of methionine synthase
Malabsorption	PA	Antibodies against IF or parietal cells
			Post gastrectomy	Decreased IF production
					Atrophic gastritis, chronic gastritis, *Helicobacter pylori*-related gastritis, achlorhydria	Decreased IF production, difficult to release B12 from food protein
					Small intestinal, bacterial overgrowth, Crohn’s disease, celiac disease, ileal resection	Decreased Absorption of B12-IF
	Chronic pancreatic exocrine insufficiency	Defective release of protease to degrade the R-binders				
			Malnutrition	Difficulties in chewing food, vegetarianism, chronic alcoholism			Difficult to release B12 from food protein, low vitamin B12 consumption

**Table 3:** Clinical manifestation of PA PA, pernicious anemia

System	Symptoms
General symptoms	Headaches Weakness Loss of appetite Weight loss due to anorexia Low-grade fever
Cardiac symptoms	Palpitation
Gastrointestinal symptoms	Painful and beefy red smooth, tongue soreness on the anterior third of the tongue, constipation, nausea, vomiting, heartburn, flatulence sense of fullness, abdominal pain with abdominal rigidity
Neurologic symptoms	Paresthesias, progressive weakness, clumsiness, ataxia, loss of proprioception, memory loss, irritability, personality changes, visual disturbances, extrapyramidal signs (e.g., dystonia, dysarthria, and rigidity), restless legs syndrome
Symptoms of thrombotic complications	Venous thrombosis due to hyperhomocysteinemia

To diagnose PA, two criteria must be met: a serum cobalamin concentration below 200 ng/L and the detection of anti-IF antibodies. While anti-IF antibodies are highly specific (100%), their sensitivity ranges from 27% to 68%, making them insufficient as a sole diagnostic marker. To enhance diagnostic accuracy, clinicians often include testing for anti-parietal cell autoantibodies. This combined approach significantly improves sensitivity, as anti-parietal cell autoantibodies are present in 85-90% of PA patients, thereby increasing the likelihood of detecting the condition [15-17]. In challenging cases of diagnosing PA, measuring the fasting serum gastrin level can be helpful. This is because autoimmune chronic atrophic gastritis, which is associated with PA, typically results in higher-than-normal gastrin levels in the blood when fasting [7].

Management of pernicious anemia

Individuals diagnosed with PA require lifelong treatment. Initially, they need intensive B12 supplementation, typically intramuscularly. In Europe, 1000 mg of hydroxocobalamin is used, while in the US, cyanocobalamin is preferred. This is administered daily or every other day for one to two weeks, followed by weekly injections for one to two months. Subsequently, patients transition to monthly injections of cyanocobalamin or injections every two to three months for hydroxocobalamin. After the initial intensive phase, patients can either continue with intramuscular injections or switch to high-dose oral B12 supplements for long-term maintenance. The most common oral supplement regimen involves taking 1000-2000 mg of cyanocobalamin daily [18].

When properly managed, PA generally has a very good prognosis. After starting treatment, patients often experience an increase in reticulocytes (immature RBCs) around day five. Within four to six weeks, the RBC count usually returns to normal levels. While blood-related improvements occur relatively quickly, neurological recovery may take more time. Interestingly, mental health symptoms like mood swings and psychosis tend to show rapid improvement [19].

According to the American Gastroenterological Association (2021), PA should be viewed as a late-stage manifestation of autoimmune gastritis. This is important because PA patients have a higher risk of developing gastric cancer. For newly diagnosed PA cases, it is recommended that patients undergo an endoscopy with topographical biopsies. This procedure serves two purposes: first, to confirm the presence of corpus-predominant atrophic gastritis, which helps in risk assessment; and second, to check for any existing gastric neoplasms, including neuroendocrine tumors [20].

## Conclusions

This case highlights the importance of considering vitamin B12 deficiency in the differential diagnosis of pancytopenia, even when severe symptoms and laboratory findings might suggest more serious conditions. Early recognition and treatment of vitamin B12 deficiency can prevent unnecessary invasive procedures and lead to rapid clinical improvement.
